# The effects of natural mating and artificial insemination using cryopreserved buck semen on reproductive performance in Alpine goats

**DOI:** 10.5194/aab-61-459-2018

**Published:** 2018-12-05

**Authors:** Dehouegnon Jerry Agossou, Nazan Koluman

**Affiliations:** Department of Animal Science, Çukurova University, Adana, 01330, Turkey

## Abstract

This study compared the effects of natural mating and artificial insemination using frozen buck semen on reproductive performance in Alpine goats. Sixty reproductive Alpine goats were grouped according to natural mating (n=30) and artificial insemination (n=30) breeding methods. Oestrus was synchronised in experimental goats using a vaginal sponge impregnated with 20 mg of progestogen FGA (fluorogestone acetate) for 11 days. At the time of sponge insertion, 150 µg of prostaglandin F${}_{2\alpha }$ (PGF${}_{2\alpha }$) analogue was injected intramuscularly. Forty-eight hours prior to vaginal sponge withdrawal, 500 IU equine chorionic gonadotropin (eCG) was injected into the animals. At the end of synchronisation protocol, goats were fertilised using frozen semen or mated with a well-performing buck (ratio: 1 male to 5 females). The pregnancy (pregnant/synchronised goats) and mortality rates (P<0.05) were higher (93 % vs. 70 %; 2 % vs. 4 %) in the naturally mated goats than the artificially inseminated group. Kids' live weight at birth was similar (3.83±0.23 and 3.15±0.11 kg) in both groups. The oestrus synchronisation followed by natural mating achieved better reproductive performance than the goats artificially inseminated using frozen semen. However, the artificially inseminated animals displayed an acceptable twinning rate.

## 1 Introduction

Optimal reproductive performance in goats is an important trait, which affects the economic profitability of farms (Yotov et al., 2016). Goat reproduction can be controlled using recently developed methods known as assisted reproductive technologies. Semen cryopreservation and artificial insemination (AI) are two methods used in the biotechnology of reproduction. Semen cryopreservation involves cooling and storing semen from elite sires in liquid nitrogen at a low temperature of -196 ${}^{\circ }$C (Sikarwar et al., 2015). This preserves the semen indefinitely, and it can be used for future AI needs. AI involves transferring semen into the reproductive tract of female animals. These methodologies enable accelerated production of genetically valuable offspring and improve reproductive performance. Nevertheless, the effectiveness of AI with frozen semen in achieving satisfactory pregnancy rates in goats remains unknown (Yotov et al., 2016). Different factors including the type of extender, interactions between cryoprotectants, cooling rate, thawing rate, and the physiological status of animals are important for successful semen cryopreservation and AI. In addition, many other factors – including nutrition; breeding season; climate conditions; parity; breed; farm; and depth of semen deposition, extender composition, or hormonal treatments – affect AI success (Baril et al., 1996; Menchaca and Rubianes, 2007; Arrebola et al., 2013). This study aimed to evaluate the effects of using frozen buck semen on the reproductive performance of artificially inseminated Alpine goats relative to naturally mated goats.

**Table 1:** Reproduction parameters of naturally and artificially (AI) inseminated Alpine goats.

Parameters	Natural	AI	P
	breeding		
Pregnancy rate (pregnant/synchronised goats) (%)	93${}^{a}$ (28/30)	70${}^{b}$ (21/30)	0.041
Litter size (no. of kids)	51	45	–
Twinning rate	$1.82\pm 0.47^{a}$	$2.14\pm 0.24^{b}$	0.040
Mortality rate (weaned/born kids) (%)	2${}^{a}$ (1/51)	4${}^{b}$ (2/45)	0.034
Litter weight at birth (kg)	3.83±0.23	3.15±0.11	0.230
Litter weight at weaning (kg)	$12.44\pm 1.25^{a}$	$11.16\pm 1.39^{b}$	0.039

## 2 Materials and methods

The experimental procedures in this study were approved by the animal welfare and ethics committee of the Faculty of Agriculture of Çukurova University. This study was carried out from August 2016 to February 2017 at the Dairy Goat Research Farm, Faculty of Agriculture, Çukurova University, located in the province of Adana, in the eastern Mediterranean region of Turkey (40 m in altitude; 36${}^{\circ }$59${}^{\prime }$ N, 35${}^{\circ }$18${}^{\prime }$ E). Sixty reproductive Alpine females (2–4 years old) and 12 Alpine bucks (3–6 years old) weighing an average of 52.95±1.21 kg were included in this study. Goats were divided into two groups of 30 goats: either naturally mated with well-performing males or artificially inseminated using frozen semen. Oestrous synchronisation was performed based on the AI timing protocol. Over 11 days, goats were synchronised using a vaginal sponge impregnated with 20 mg of progestogen FGA (fluorogestone acetate, Chronogest CR, Intervet, France). Upon insertion of the sponge, 150 µg of prostaglandin F${}_{2\alpha }$ (PGF${}_{2\alpha }$) analogue was injected intramuscularly. At day 9 (i.e. 48 h prior to vaginal sponge withdrawal, 500 IU equine chorionic gonadotropin (eCG) was administrated to the animals. To detect the does in standing oestrus, a teaser buck equipped with an apron was introduced into the experimental groups twice per day: in the morning at 08:00–09:00 LT and evening at 17:00–18:00 LT. Within 48 to 52 h following the synchronisation protocol, goats detected as standing in oestrus were inseminated with frozen–thawed semen following the intrauterine AI methods described by Tsuma et al. (2015). Only semen with a high mobile spermatozoa score (>55 % mobile spermatozoa), sperm motility (3.0 or above), and concentration of $55\times 10^{6}$ spermatozoa ml${}^{-1}$ was used. For naturally mated goats, a ratio of 1 male to 5 females was applied. All females were doubly mated within 48 h following oestrus detection. Eight hours after the first mating, they were mated for the second time with the same buck. Pregnancy was confirmed by trans-abdominal ultrasound (5.0 MHz) 35–40 days after insemination.

Reproductive parameters including pregnancy, twinning, and mortality rate were subjected to analysis of frequency (percentages) calculations. An analysis of variance was performed concerning the other reproductive parameters (live weight of kids at birth). Differences between treatment group means were tested using Chi-square test at a level of 5 % using the program SPSS 20.

## 3 Results and discussion

Data on reproductive parameters of Alpine goats after natural mating and artificial insemination with frozen semen following oestrus synchronisation are given in Table 1. The pregnancy rates for naturally mated and artificially inseminated goats were 93 % and 70 %, respectively. The pregnancy rate in naturally bred goats was higher than AI goats. The pregnancy rate and number of newly born kids are the most important indicators with which to evaluate reproductive performance in animal agriculture (Yotov et al., 2016). The calculated pregnancy rate in naturally bred goat groups was higher than the 85.7 % recorded by Fonseca et al. (2005) in Alpine goats treated with intravaginal sponges (MAP, 60 mg) for 9 days plus 200 IU eCG and 22.5 µg d-cloprostenol 24 h before sponge removal and similar to the 93.44 % reported by Duričić et al. (2012) in Boer goats reared in a semi-intensive environment in Croatia. The results show that the mortality rate (4 % vs. 2 %) was significantly higher in AI goats than in the natural breeding group. A similar trend was observed for the twinning rate (2.14 vs. 1.82), for which the obtained values were significantly different among treatment groups.

Several reasons may justify the low pregnancy and kidding rate obtained in the AI group relative to natural breeding. Goats must be inseminated 12 h after the onset of oestrus and 5–10 h before the expected ovulation (Lehloenya et al., 2005). In this trial, AI was performed 48–52 h after withdrawal of the vaginal sponge, i.e. 24–28 h after the onset of oestrus was detected. The high pregnancy rate from natural mating could be ascribed to the protective role of fresh semen plasma.

The average live weight at birth and weaning was 3.83±0.23 and 3.15±0.11 kg for natural breeding, and 12.44±1.25 and 11.16±1.39 kg for AI, respectively. Kids born in the natural breeding group were heavier than those in the AI group. Many previous studies have established a negative correlation between birth weight and litter size; birth weight decreases as litter size increases (Freetly and Leymaster, 2004; Mellado et al., 2011). Therefore, the large litter size observed in this study explains the low birth weight recorded in the AI group. In addition, Lehloenya et al. (2005) argue that kids from multiple births tend to be weaker at birth than single kids. This weakness usually leads to a low survival and high mortality rate for multiple kids as a result of physiological starvation in the uterus and birth with lower energy reserves. This supports the higher mortality rate observed in the AI group in the present study.

## 4 Conclusion

This study shows that oestrus synchronisation followed by natural mating was more effective (high pregnancy and survival rates and birth weight) than the use of AI after oestrus synchronisation in goats. However, AI animals demonstrated an acceptable twinning rate. Several driving factors have been identified as potentially affecting reproduction and milk production in dairy goats.

## Data Availability

Data from this research study can be obtained by request from the corresponding author.
